# Artificial Intelligence in Managing Spasticity with Botulinum Toxin Type A—Insights from an Exploratory Pilot Investigation: The AIMS Study

**DOI:** 10.3390/toxins17120573

**Published:** 2025-11-27

**Authors:** Mirko Filippetti, Rita Di Censo, Lyria Arcari, Maria Concetta Schiavariello, Marco Battaglia, Salvatore Facciorusso, Stefania Spina, Laura Antonucci, Andrea Santamato, Alessio Baricich, Nicola Smania, Alessandro Picelli

**Affiliations:** 1 Section of Physical and Rehabilitation Medicine, Department of Neurosciences, Biomedicine and Movement Sciences, University of Verona, 37134 Verona, Italy; mirko.filippetti@univr.it (M.F.); rita.dicenso@univr.it (R.D.C.); lyria.arcari@studenti.univr.it (L.A.); mariaconcetta.schiavariello@studenti.univr.it (M.C.S.); nicola.smania@univr.it (N.S.); 2 Canadian Advances in Neuro-Orthopedics for Spasticity Consortium (CANOSC), Kingston, ON K7K 1Z6, Canada; 3 Neurorehabilitation Unit, Department of Neurosciences, University Hospital of Verona, 37126 Verona, Italy; 4 Department of Health Sciences, University of Piemonte Orientale, 28100 Novara, Italy; marco.battaglia@uniupo.it; 5 Rehabilitation Unit, IRCCS Humanitas Research Hospital, 20089 Rozzano, Italy; alessio.baricich@hunimed.eu; 6 Spasticity and Movement Disorders “ReSTaRt”, Physical Medicine and Rehabilitation Section, Department of Medical and Surgical Sciences, University of Foggia, 71122 Foggia, Italy; s.facciorusso89@gmail.com (S.F.); spinastefania.ss@gmail.com (S.S.); andrea.santamato@unifg.it (A.S.); 7 Fondazione IRCCS Don Carlo Gnocchi ONLUS, 50143 Florence, Italy; lantonucci@dongnocchi.it; 8 Department of Biomedical Sciences, Humanitas University, 20090 Pieve Emanuele, Italy

**Keywords:** botulinum toxins, machine learning, muscle spasticity

## Abstract

This study aimed to explore the potential role of artificial intelligence in optimizing botulinum toxin type A treatment for spasticity and to evaluate its alignment with expert clinical decisions. A comparative analysis was conducted using thirty hypothetical clinical cases involving individuals with spasticity resulting from various neurological conditions. Five rehabilitation physicians, each with more than five years of experience, participated in the study. An artificial intelligence model trained on scientific literature and clinical guidelines generated treatment recommendations, including target muscles and dosages, which were compared with those proposed independently by the physicians. The primary outcome was the level of agreement in muscle selection and dosage. The model demonstrated consistency and adherence to guidelines but showed limited adaptability in complex presentations, such as an adducted thigh and equinovarus foot. It generally recommended lower dosages and differed significantly from physicians in both muscle selection and treatment strategies. Artificial intelligence shows promise as a clinical support tool in spasticity management, offering standardized and reproducible recommendations. However, its limited capacity to interpret clinical subtleties currently restricts its practical application. Future models should integrate multimodal clinical data and real-time clinician feedback to better emulate expert decision-making processes.

## 1. Introduction

Artificial intelligence (AI), a technology designed to perform tasks that typically require human intelligence (such as learning, reasoning, and decision-making), is becoming increasingly prevalent in everyday applications, with its role in the medical field expanding rapidly. AI has the potential to impact healthcare by enhancing and supporting clinical decision-making through generative models and machine learning tools [1].

To date, AI has been integrated into various areas of medicine, driving significant advancements in diagnosis, treatment planning, and rehabilitation [2,3,4,5]. Machine learning algorithms are capable of processing large datasets to identify patterns and predict disease progression, thereby enabling more personalized treatment strategies [6]. In neurology, AI has demonstrated potential in stroke detection, lesion segmentation, and outcome prediction, contributing to improved clinical decision-making [7]. Despite the rapid expansion of AI into new medical domains, its application in the management of spasticity remains largely unexplored. Given the complexity and continuous evolution of AI algorithms, their potential to support spasticity assessment and optimize treatment strategies warrants further investigation.

Spasticity is a common consequence of upper motor neuron lesions due to some different etiologies [8,9,10]. Current spasticity management relies on a multidisciplinary approach that includes both pharmacological and non-pharmacological interventions. Among pharmacological treatments, botulinum toxin type A (BoNT/A) injections have been shown to be safe and effective for focal spasticity, with robust evidence supporting their use in stroke, traumatic brain injury, spinal cord injury, multiple sclerosis, and cerebral palsy [11,12,13,14,15]. However, spasticity presents with a highly heterogeneous clinical profile, involving various types of muscle overactivity (such as spasticity, spastic dystonia, co-contraction, associated reactions, and clonus) as well as overlapping features of spastic myopathy [16,17]. This variability poses significant diagnostic and therapeutic challenges for physicians.

Given its clinical relevance and substantial impact on quality of life (contributing to pain, contractures, and functional impairments that limit independence in daily activities), optimizing spasticity management is essential for improving patient outcomes. Integrating AI into spasticity management could have a transformative effect on patient care by providing objective assessment tools, improving the identification of spasticity patterns, and enhancing rehabilitation outcomes. Specifically, in the context of spasticity treatment, AI holds promise for refining patient-specific therapeutic strategies, particularly by improving BoNT/A administration based on individual clinical presentations. The *A*rtificial *I*ntelligence in *M*anaging *S*pasticity (*AIMS*) study wants to explore the potential impact of generative AI technologies in optimizing BoNT/A therapy for patients with spasticity, addressing current limitations and future directions.

## 2. Results

Following the definition of treatment patterns based on consensus among the five rehabilitation physicians, each clinician provided a recommended dosage for each identified muscle, tailored to the corresponding clinical case. Table 1 summarizes the mean and standard deviation of the proposed doses for each spasticity pattern per specialist, along with the overall mean and the corresponding values generated by the AI model.

Given the non-normal distribution of the data, median values and interquartile ranges were also calculated and are presented in Table 2. Both tables include the frequency with which each spasticity pattern was selected for treatment by the specialists and by the AI model.

Descriptive analyses revealed that the dosages proposed by the specialists (both mean and median) were generally higher than those suggested by the AI model. The only exception was the “flexed knee” pattern, which the AI selected for treatment in only 1 of 30 cases, compared to 4 instances among the specialists. Table 3 presents the results of the Wilcoxon signed-rank test comparing each specialist’s median dosage to the overall median across patterns. The analysis showed that some specialists consistently proposed significantly higher doses for specific spasticity patterns.

In particular, significant deviations were observed for: Specialist 1 and Specialist 4 in the “adducted thigh” pattern; Specialist 2 in the “flexed wrist” and “flexed toes” patterns; Specialist 5 in the “flexed elbow” and “thumb-in-palm” patterns. Only Specialist 3 showed no significant deviation from the overall median in any pattern. Table 4 reports the comparisons between the AI-generated dosages and those proposed by each individual specialist, as well as the aggregated group. Statistically significant differences emerged in the “flexed wrist,” “adducted thigh,” and “equinovarus foot” patterns, where the AI consistently proposed lower mean and median dosages. In pairwise comparisons, the “adducted thight” pattern differed significantly in 4 out of 5 cases, and the “equinovarus foot” pattern in 3 out of 5. A single significant difference was observed for the “flexed fingers” pattern (AI vs. Specialist 2).

A qualitative analysis of treatment plans further highlighted discrepancies between the AI model and the human specialists. Across the 30 clinical cases, a total of 83 divergences were identified. The main categories of mismatch and their relative frequencies are reported in Table 5. Notably, only 3 of the 30 cases demonstrated a high level of agreement, characterized by substantial overlap in target muscle selection between the AI model and all five specialists.

## 3. Discussion

The AIMS study provides a comprehensive evaluation of the potential and limitations of AI in supporting clinical decision-making for the management of spasticity with BoNT/A. The findings consistently show that the AI model proposed lower dosages and often diverged from expert consensus in muscle selection and treatment prioritization. These results highlight both the promise of AI as a decision-support tool and the critical need for human oversight in complex, individualized clinical scenarios.

Quantitative analyses confirmed that the AI model tended to recommend significantly lower doses for several clinically relevant spasticity patterns. The Wilcoxon signed-rank test revealed statistically significant differences between AI and specialists in treating the adducted thigh, equinovarus foot, and flexed wrist patterns (Table 5). This conservative dosing bias likely stems from the model’s training data, which may have emphasized safety and underrepresented complex or borderline cases.

Inter-individual variability among human experts was also evident. For instance, Specialist 1 and Specialist 4 proposed higher doses for the adducted thigh, while Specialist 2 and Specialist 5 deviated significantly in treating the flexed wrist, flexed toes, and thumb-in-palm patterns. This variability underscores the subjective and personalized nature of BoNT/A treatment planning, which often relies on clinical experience and contextual judgment rather than standardized protocols.

Qualitative analysis of the results of this study identified 83 discrepancies between AI-generated and expert consensus treatment plans, categorized into eight types (Table 6). The most frequent was overtreatment within the same pattern (20.5%), where AI recommended injecting multiple muscles without prioritization. In contrast, clinicians adopted a more selective approach, balancing therapeutic goals, spasticity severity, and functional trade-offs. Other common discrepancies included omission of relevant muscles (16.9%) and failure to consider contextual factors (15.7%), such as pain, residual function, or compensatory strategies, areas where AI currently lacks interpretive flexibility.

Additional issues included substitution of target muscles (10.8%), symmetrical treatment despite asymmetry (9.6%), and undertreatment of the upper limb (9.6%). These findings suggest that while AI can process structured data effectively, it lacks the contextual awareness and nuanced reasoning that characterize expert clinical judgment. For example, a mildly spastic limb may be intentionally left untreated if it contributes to functional compensation, an assessment beyond current AI capabilities.

Despite these differences between AI and the Panel of experts, the AIMS study also highlights the potential utility of AI in clinical practice. AI can serve as a standardized decision-support tool, particularly beneficial for less experienced clinicians. By offering evidence-based initial recommendations, AI may reduce variability in care and support early injectors in building clinical confidence. Furthermore, its ability to synthesize patient data, spasticity classification scales, and gait descriptors in real time enables rapid generation of preliminary treatment plans, improving workflow efficiency in busy outpatient settings.

To ensure safe and effective integration into clinical workflows, the AI limitations highlighted must be addressed. The AI model’s reliance on structured data and inability to interpret subjective variables constrain its applicability in complex cases. Future models should incorporate multimodal data sources, such as electromyographic signals, three-dimensional motion capture, inertial sensor metrics, and validated patient-reported outcomes, to approximate the holistic understanding developed by experienced clinicians.

Transparency and safety frameworks are equally important. AI systems should provide confidence scores and flag low-certainty recommendations or areas with sparse training data. These features would prompt clinicians to apply additional scrutiny and seek further input when necessary. Maintaining an auditable record of AI-human interactions would also support retrospective analyses and continuous model refinement.

In the field of neurorehabilitation, recent advances in research provide a comprehensive picture of how artificial intelligence (AI) may contribute to the assessment, management, and delivery of rehabilitation services for patients with neurological impairments. This can be considered evidence highlighting that AI-based models are increasingly employed to refine diagnosis, stratify disease severity, and predict functional outcomes across conditions such as stroke, Parkinson’s disease, neuromuscular disorders, and cognitive decline. These systems integrate heterogeneous data streams, including wearable sensor outputs, accelerometry, electromyographic signals, sleep-related physiological metrics, and computer-vision–based gait analysis, to capture subtle fluctuations in motor performance and detect clinically relevant patterns that may not be easily observed through conventional examination [18].

Furthermore, AI-driven robotic platforms, brain–computer interfaces, and machine learning–based decision support systems enable more intensive, adaptive, and high-dose rehabilitation programs by dynamically adjusting task difficulty and providing real-time feedback based on patient performance. Such decision support systems have shown particular promise in guiding clinicians during stroke rehabilitation assessment, allowing for more precise and personalized intervention planning [19]. Beyond clinic-based applications, AI also facilitates home-based and remotely supervised rehabilitation pathways, enabling continuous monitoring, early detection of functional decline, and delivery of tailored exercise programs to support long-term recovery [20].

Finally, recent systematic reviews emphasize the transformative potential of AI across rehabilitation services, highlighting opportunities to optimize workflow, enhance patient engagement, and integrate multimodal data streams into evidence-based care models [21].

Looking ahead, adaptive learning paradigms may offer a promising direction. By incorporating real-world outcomes (such as treatment efficacy, functional improvements, and patient satisfaction), AI systems can evolve and recalibrate their recommendations based on empirical evidence. Collaborative networks that pool de-identified clinical data across institutions could enhance generalizability and reduce bias. Ultimately, AI should be seen not as a replacement for clinical expertise, but as a dynamic partner that augments human judgment and elevates the standard of care.

Several limitations of this study should be acknowledged. First, the AI model was trained exclusively on structured clinical data and scientific literature, which may limit its generalizability to the nuanced presentations of spasticity encountered in real-world practice. This likely contributed to the model’s conservative dosing tendencies, especially in high-impact patterns. Second, the algorithm lacked access to contextual information, such as prior treatment history, patient preferences, and therapeutic rationale, that often guides expert decisions. Third, while the use of 30 hypothetical clinical cases allowed for controlled comparison, the limited sample size may restrict generalizability. Real-world validation in larger, more diverse populations is needed. Fourth, it is impossible to determine whether any features specific to one of the 30 analyzed cases were inadvertently introduced during the model’s training process, which could have compromised the model’s independence of judgment. Fifth, because the iterative phase with the model was not stored during the training phase, this limits the full reproducibility of the study. Fifth, the observed variability among the five specialists reflects the subjective nature of spasticity management, an aspect current AI models are not yet equipped to replicate. A further key limitation of our analysis is that multiple muscles within the same clinical case were treated as independent observations in the Wilcoxon signed-rank tests, which may have inflated statistical significance. In addition, the ‘reference median’ used in the one-sample tests was an aggregated descriptive measure rather than a true population parameter, potentially compromising the inferential validity of these analyses. For these tests, the reference value was calculated as the overall median of the specialists’ median dosages across all spasticity patterns. This approach was intended to provide a central tendency benchmark; however, we acknowledge that it does not represent a formal population parameter and may limit the statistical validity of the test. Therefore, these results should be interpreted as exploratory rather than confirmatory.

Future development of AI tools in this field should prioritize expanding training datasets to include more diverse and complex clinical scenarios. Incorporating multimodal data, such as biomechanical analyses, electromyographic signals, gait metrics, and patient-reported outcomes, may enhance the clinical relevance and accuracy of AI-generated recommendations. Additionally, hybrid systems that integrate human oversight and iterative feedback (i.e., human-in-the-loop models) represent a promising path for safe and effective AI implementation in rehabilitation medicine. Due to their relevance, future research should also explore the ability of AI to support the multimodal management of patients with spasticity and to advise on the most appropriate adjunctive therapies.

In conclusion, while AI-based systems offer a standardized and reproducible approach to treatment planning, they currently lack the flexibility and contextual sensitivity of expert human judgment. The observed discrepancies in dosage and muscle selection highlight the limitations of AI in complex, individualized rehabilitation scenarios. These findings reinforce the current impossibility of AI to replace clinical expertise. Rather than replacing the clinician’s role, in the near future, it cannot be ruled out that artificial intelligence could play a supporting role in the clinical decision-making process. Ideally, AI should function within a human-in-the-loop framework, where its strengths in data synthesis and pattern recognition augment, but do not override, clinical decision-making. Such integration can enhance consistency, support less experienced clinicians, and improve workflow efficiency, particularly in resource-limited settings. By evolving toward systems that reflect the complexity of real-world decision-making, AI can become a valuable partner in advancing personalized rehabilitation care.

## 4. Materials and Methods

The AIMS study is an exploratory pilot study that compared treatment recommendations generated by an AI model with those provided by five rehabilitation physicians. Each of them has a minimum of five years of experience in the management of spasticity, holds an in-depth knowledge of the characteristics of the three pharmaceutical formulations in Italy available (OnabotulinumtoxinA, AbobotulinumtoxinA, and IncobotulinumtoxinA), and regularly uses all of them in outpatient settings following standardized procedures. All five practitioners operate under the same regulations established by the Italian healthcare system.

To facilitate this comparison, a set of 30 hypothetical clinical cases was developed by three independent senior physiatrists, based on real medical records of patients with spasticity secondary to stroke, multiple sclerosis, cerebral palsy, and brain injury. To each case, modifications and additional descriptive elements were applied both to make the case more suitable for the study’s purpose and to ensure that it could not be traced back to real individuals, in accordance with ethical standards. Each case included detailed information on the underlying pathology, functional status of the upper and lower limbs, gait characteristics, and additional clinical aspects such as pain and the use of orthoses or assistive devices.

Spasticity was described using established classification patterns for both upper and lower limbs [22,23,24,25], along with assessments of passive range of motion, the Modified Ashworth Scale [26,27], and the Tardieu Scale (including both score and angle) for evaluating spastic muscle overactivity [28]. The clinical scenarios incorporated a range of variables, including gender, etiology, and anatomical distribution of spasticity (proximal and distal muscles of the upper and lower limbs), ensuring a representative spectrum of clinical presentations.

The AI model employed was OpenAI o1, a reflective generative pre-trained transformer (GPT), released to ChatGPT users on 5 December 2024 [29]. Compared to GPT-4o, OpenAI o1 is designed to engage in deeper reasoning prior to response generation, enhancing its performance in complex decision-making, scientific reasoning, and programming tasks [30].

Before submitting the clinical cases to the AI model, a structured training phase was conducted. During this phase, the model was provided with a curated selection of key literature on spasticity and BoNT/A treatment. A rehabilitation physician engaged in iterative discussions with the model, focusing on the various formulations of BoNT/A and their approved indications; guidelines; interpretation and application of spasticity assessment scales; analysis of gait descriptions; and the correlation between spasticity patterns and appropriate muscle targets. When knowledge gaps were identified, additional literature was supplied to refine the model’s reasoning and ensure a robust understanding of the clinical context.

Following training, both the AI model and the five specialists were asked to respond to the same questions for each of the 30 clinical cases as follows:

(a) Based on the clinical information provided, which muscles should be targeted for BoNT/A treatment?

(b) Considering the different formulations available on the market (i.e., onabotulinumtoxinA, abobotulinumtoxinA, and incobotulinumtoxinA), which treatment approach involving BoNT/A (such as the choice of formulation and dosage) would you recommend for each targeted muscle?

As to the last question, to ensure consistency in dosage comparisons, abobotulinumtoxinA units were converted using a 3:1 ratio relative to onabotulinumtoxinA and incobotulinumtoxinA [31]. Each case was presented to the AI model individually and sequentially, without feedback between responses, to minimize bias and ensure independent decision-making. Responses were analyzed using descriptive statistical methods. Cross-tabulations were created in Excel (Microsoft, Redmond, WA, USA) to compare the AI-generated recommendations with those of the specialists.

### 4.1. Description of the GPT-o1 Algorithm (Self-Generated by AI)

The GPT-4-based model used in the AIMS study is a natural language processing system employing transformer architecture to analyze and generate human-like text. Trained on a broad corpus of data (including scientific literature, clinical guidelines, and general knowledge), it is capable of providing insights across various domains, including medicine and scientific research. Its strengths include contextual understanding and coherent response generation, making it a useful tool for knowledge synthesis, hypothesis generation, and clinical decision support.

The model’s versatility allows it to process large volumes of information and integrate data from multiple sources to produce informed recommendations. In scientific contexts, this facilitates rapid literature reviews, identification of data trends, and evidence-based decision-making. Nonetheless, limitations persist, including potential misinterpretation of complex queries and the perpetuation of biases present in training data. Moreover, the model lacks direct experiential knowledge, which restricts its applicability in contexts requiring sensory perception. Its outputs should therefore be validated and used to complement, not replace, human expertise. Future integration of multimodal data may further enhance its accuracy and expand its clinical utility.

### 4.2. Consensus Among Rehabilitation Physicians

Each of the five rehabilitation physicians independently answered the same questions posed to the AI model. Initial recommendations were provided without external influence or discussion. Responses were compiled and analyzed to determine the level of agreement.

The first level of analysis focused on muscle selection. A muscle was considered definitive if selected by at least four out of five specialists and excluded if chosen by only one. Muscles identified by two or three rehabilitation physicians underwent a second round of discussion, during which all five experts reviewed the cases collectively to reach a consensus of at least four votes. If a muscle received only two votes and failed to gain additional support, it was excluded. If a muscle with two votes increased to three, or if a muscle with three votes failed to reach four, a sixth specialist was consulted. If the muscle then received a fourth vote, it was included; otherwise, it was excluded (see Table 6).

Once the definitive muscle profile was established for each case, the five specialists were asked to recommend a dosage for each selected muscle. The total, mean, minimum, and maximum doses were calculated and compared with the AI-generated dosages.

This allows one to establish a realistic treatment plan and evaluate whether the AI-proposed treatment is equally realistic, potentially effective, and within the limits defined by current guidelines.

### 4.3. Statistical Analysis

Descriptive statistics (mean, standard deviation, median, and interquartile ranges) were calculated for the variable “units administered.” The Shapiro–Wilk test was used to assess normality. As the data were not normally distributed, non-parametric methods were applied. Pairwise comparisons between the AI model and each individual specialist were conducted using the Wilcoxon signed-rank test for paired samples, assessing differences in the median number of units administered per target muscle. These analyses were performed at the muscle level, acknowledging that multiple muscles within a single case are not independent observations. Therefore, the results should be interpreted as exploratory and hypothesis-generating rather than confirmatory. To mitigate inflated significance, we also calculated case-level medians and repeated the analysis as a sensitivity check, which yielded consistent trends. All analyses were performed using SPSS software version 27.0 for Macintosh (IBM Corp., Armonk, NY, USA), with statistical significance set at *p* ≤ 0.05.

The independent variable in this study was the source of treatment recommendations (artificial intelligence model vs. rehabilitation physicians). The dependent variables were: (i) the selection of target muscles for BoNT/A injection and (ii) the recommended dosage for each muscle. Additional descriptive variables included spasticity pattern, anatomical distribution (upper vs. lower limb), and clinical context (e.g., pain, functional status).

Constraints applied in the study included: (a) all clinical cases were hypothetical and anonymized to ensure ethical compliance; (b) physicians had a minimum of five years of experience in spasticity management and routinely used all three BoNT/A formulations available in Italy; (c) dosage recommendations were standardized by converting abobotulinumtoxinA units to onabotulinumtoxinA/incobotulinumtoxinA equivalents using a 3:1 ratio; and (d) AI responses were generated without iterative feedback between cases to avoid bias. Statistical analyses were limited to non-parametric methods due to non-normal data distribution.

## Figures and Tables

**Table 1:** Number (n) of muscles and mean dose of botulinum toxin type A for each upper and lower limb spasticity pattern of each rehabilitation physician and artificial intelligence.

Pattern	n	Physician 1	Physician 2	Physician 3	Physician 4	Physician 5	All	n	Artificial Intelligence
Adducted shoulder	8	81.21 ± 39.82	80.83 ± 25.49	70.21 ± 16.58	73.74 ± 38.69	68.74 ± 23.22	74.98 ± 5.84	1	40.00 ± n.c.
Flexed elbow	11	80.29 ± 59.67	67.58 ± 28.44	76.51 ± 34.72	81.47 ± 43.05	61.51 ± 29.86	70.90 ± 8.62	7	62.86 ± 29.84
Flexed wrist	6	66.63 ± 29.83	56.67 ± 8.16	83.33 ± 25.82	64.13 ± 16.84	73.88 ± 31.17	64.98 ± 9.94	6	41.67 ± 17.22
Flexed fingers	9	77.78 ± 38.44	76.67 ± 37.16	51.36 ± 34.07	50.90 ± 19.65	70.00 ± 31.22	73.89 ± 13.31	7	52.14 ± 28.85
Thumb in palm	5	43.32 ± 9.15	46.00 ± 8.94	26.32 ± 6.69	37.98 ± 8.37	22.00 ± 4.47	32.66 ± 10.53	4	21.25 ± 2.50
Adducted thigh	12	65.96 ± 10.33	87.48 ± 7.55	88.18 ± 11.50	55.53 ± 27.73	84.83 ± 28.34	75.40 ± 14.81	13	36.41 ± 13.76
Flexed knee	4	75.03 ± 16.65	86.65 ± 9.03	75.00 ± 20.41	137.45 ± 79.81	75.84 ± 30.47	75.44 ± 5.69	1	140.00 ± n.c.
Extended knee	4	68.73 ± 27.55	88.33 ± 14.53	95.83 ± 8.35	101,65 ± 25.20	76.67 ± 25.17	72.70 ± 13.53	6	70.83 ± 24.58
Equinovarus foot	35	189.85 ± 68.77	176.75 ± 63.48	164.00 ± 56.79	179.80 ± 64.89	169.30 ± 84.13	179.58 ± 9.95	39	145.64 ± 48.71
Flexed toes	9	45.33 ± 17.22	72.50 ± 27.12	63.89 ± 22.05	56.26 ± 26.21	53.81 ± 15.29	49.57 ± 8.04	6	45.83 ± 18.82
Striatal toe	4	37.48 ± 8.35	31.65 ± 1.91	28.75 ± 2.50	37.48 ± 8.35	29.15 ± 9.60	33.32 ± 4.32	5	18.99 ± 6.94

*n.c.: not calculated by AI*.

**Table 2:** Number (n) of muscles and median dose of botulinum toxin type A for each upper and lower limb spasticity pattern of each rehabilitation physician and artificial intelligence.

Pattern	n	Physician 1	Physician 2	Physician 3	Physician 4	Physician 5	All	n	Artificial Intelligence
Adducted shoulder	8	75.00 (54.17; 75.00)	73.33 (66.67; 83.30)	68.33 (54.16; 81.22)	56.65 (50.00; 91.65)	60.00 (52.50; 79.12)	68.33 (60.00; 73.33)	1	40.00 (40.00; 40.00)
Flexed elbow	11	75.00 (33.30; 75.00)	66.67 (50.00; 66.67)	75.00 (50.00; 75.00)	66.60 (66.60; 80.00)	50.00 (50.00; 60.00)	66.67 (66.60; 75.00)	7	50.00 (40.00; 100.00)
Flexed wrist	6	66.60 (33.30; 100.00)	55.00 (50.00; 62.50)	100.00 (50.00; 100.00)	66.60 (47.50; 77.90)	80.00 (38.32; 102.50)	66.60 (66.60; 80.00)	6	50.00 (20.00; 52.50)
Flexed fingers	9	75.00 (41.65; 112.50)	66.67 (50.00; 116.67)	50.00 (25.00; 80.00)	43.30 (36.65; 72.50)	60.00 (45.00; 105.00)	60.00 (50.00; 66.67)	7	40.00 (30.00; 80.00)
Thumb in palm	5	50.00 (33.30; 50.00)	50.00 (40.00; 50.00)	25.00 (20.00; 33.30)	33.30 (31.65; 46.65)	20.00 (20.00; 25.00)	33.30 (25.00; 50.00)	4	20.00 (20.00; 23.75)
Adducted thigh	12	66.65 (54.15; 75.00)	83.30 (83.30; 95.82)	83.30 (83.30; 100.00)	48.30 (33.30; 63.27)	83.30 (66.60; 100.00)	83.30 (66.65; 83.30)	13	30.00 (25.00; 50.00)
Flexed knee	4	66.70 (66.70; 91.67)	83.30 (80.82; 95.82)	75.00 (56.25; 93.75)	116.60 (74.95; 220.80)	66.67 (54,16; 106.66)	75.00 (66.70; 83.30)	1	140.00 (140.00; 140.00)
Extended knee	4	70.80 (41.62; 93.75)	91.65 (73.32; 100.00)	100.00 (87.47; 100.00)	110.00 (74.95; 120.00)	73.30 (54,15; 95.00)	91.65 (73.30; 100.00)	6	62.50 (50.00; 100.00)
Equinovarus foot	35	200.00 (133.30; 233.30)	150.00 (133.30; 216.67)	166.67 (125.00; 200.00)	170.00 (133.30; 240.30)	166.60 (100,00; 217.45)	166.67 (166.60; 170.00)	39	150.00 (100.00; 200.00)
Flexed toes	9	33.30 (33.30; 66.60)	66.67 (50.00; 95.00)	50.00 (50.00; 87.50)	50.00 (33.30; 69.90)	50.00 (45,00; 70.00)	50.00 (50,00; 50.00)	6	50.00 (25.00; 56.25)
Striatal toe	4	33.30 (33.30; 45.82)	31.65 (30.00; 33.30)	30.00 (26.25; 30.00)	33.30 (33.30; 45.82)	30.00 (19,95; 37.50)	31.65 (30,00; 33.30)	5	20.00 (12,48; 25.00)

**Table 3:** Wilcoxon analysis of the median botulinum toxin type A dose of each rehabilitation physician versus all physicians for each pattern of spasticity.

Pattern	n	Physician 1 Versus All	Physician 2 Versus All	Physician 3 Versus All	Physician 4 Versus All	Physician 5 Versus All
Adducted shoulder	8	0.570	0.260	0.779	0.673	0.481
Flexed elbow	11	0.586	0.332	0.857	0.784	**0.041**
Flexed wrist	6	0.458	**0.043**	0.105	0.715	0.462
Flexed fingers	9	0.212	0.399	0.776	0.138	0.497
Thumb in palm	5	0.083	0.066	0.102	0.285	**0.034**
Adducted thigh	12	**0.003**	0.083	0.054	**0.029**	0.943
Flexed knee	4	0.705	0.066	1.000	0.144	0.713
Extended knee	4	0.144	0.705	0.317	0.269	0.144
Equinovarus foot	35	0.143	0.851	0.750	0.351	0.950
Flexed toes	9	0.087	**0.046**	0.102	0.733	0.223
Striatal toe	4	0.059	1.000	0.059	0.059	0.461

**Table 4:** Wilcoxon analysis of the median botulinum toxin type A dose between each and all physicians versus artificial intelligence for each pattern of spasticity.

Pattern	n	Artificial Intelligence Versus Physician 1	Artificial Intelligence Versus Physician 2	Artificial Intelligence Versus Physician 3	Artificial Intelligence Versus Physician 4	Artificial Intelligence Versus Physician 5	Artificial Intelligence Versus All
Adducted shoulder	8	n.c.	n.c.	n.c.	n.c.	n.c.	n.c.
Flexed elbow	11	0.553	0.866	0.866	0.735	0.752	0.734
Flexed wrist	6	0.066	0.102	0.066	0.068	0.068	**0.041**
Flexed fingers	9	0.068	0.068	0.500	0.345	0.068	0.498
Thumb in palm	5	0.066	0.066	0.180	0.068	0.655	0.059
Adducted thigh	12	**0.026**	**0.010**	**0.011**	0.246	**0.017**	**0.016**
Flexed knee	4	n.c.	n.c.	n.c.	n.c.	n.c.	n.c.
Extended knee	4	0.593	0.285	0.180	0.109	0.655	0.458
Equinovarus foot	35	**0.002**	**0.026**	0.180	**0.009**	0.215	**0.030**
Flexed toes	9	0.892	**0.043**	0.063	0.080	0.080	0.564
Striatal toe	4	0.066	0.066	0.066	0.068	0.144	0.066

*n.c.: not calculated*.

**Table 5:** Type of discrepancy and its frequency between rehabilitation physicians’ consensus treatment and artificial intelligence.

Type of Discrepancy	Frequency	Percentage
Treatment of more muscles of the same pattern	17	20.48
Omission of clinically relevant muscles	14	16.87
Ignoring clinical context (e.g., pain, functionality)	13	15.67
Substitution of target muscles	9	10.84
Symmetrical treatment despite asymmetry	8	9.64
Under-treatment of upper limb	8	9.64
Overtreatment of functionally preserved areas	7	8.43
Omission of shoulder muscles	7	8.43
Total	83	100

**Table 6:** Consensus rules for rehabilitation physicians.

Number of Specialists Selecting Muscle	Decision
**First round**	
1 out of 5	Discarded
2 or 3 out of 5	Discussed in the second round
4 or 5 out of 5	Definitive muscle target
**Second round**	
2 out of 5	If no additional support, discarded
	If only one additional support, sixth specialist was consulted; if yes, definitive muscle target; if not, discarded
3 out of 5	If fourth preference obtained, definitive muscle target
	If no further preference obtained, sixth specialist was consulted. If yes, definitive muscle target; if no, discarded

## Data Availability

The original contributions presented in this study are included in the article. Further inquiries can be directed to the corresponding author.

## Abbreviations

The following abbreviations are used in this manuscript:

AI	Artificial Intelligence
BoNT/A	Botulinum toxin type A
